# Health and aging trajectories: shared and competing risks and resiliencies for chronic diseases associated with aging. A NIH-wide workshop

**DOI:** 10.3389/fpubh.2025.1462217

**Published:** 2025-05-01

**Authors:** Ilsa I. Rovira, Arya Biragyn, LaVerne L. Brown, Zorina S. Galis, Malgorzata Klauzinska, Svetlana E. Kotliarova, Janine M. Simmons, Anil Wali, Dan Xi, Ronit I. Yarden, Gabriela Riscuta

**Affiliations:** ^1^Division of Cardiovascular Sciences, National Heart, Lung, and Blood Institute (NHLBI), National Institutes of Health (NIH), Bethesda, MD, United States; ^2^Laboratory of Molecular Biology and Immunology (LMBI), Immunoregulation Section, National Institute on Aging (NIA), National Institutes of Health (NIH), Baltimore, MD, United States; ^3^Resilience and Health Studies Program, National Institutes of Health (NIH), Office of Dietary Supplements (ODS), Bethesda, MD, United States; ^4^Division of Cancer Biology, National Cancer Institute (NCI), National Institutes of Health (NIH), Bethesda, MD, United States; ^5^Division of Neuroscience, National Institute of Neurological Disorders and Stroke (NINDS), National Institutes of Health (NIH), Bethesda, MD, United States; ^6^Office of Behavioral and Social Sciences Research (OBSSR), National Institutes of Health (NIH), Bethesda, MD, United States; ^7^Center to Reduce Cancer Health Disparities, National Cancer Institute (NCI), National Institutes of Health (NIH), Bethesda, MD, United States; ^8^Division of Cancer Treatment and Diagnosis, National Cancer Institute (NCI), National Institutes of Health (NIH), Bethesda, MD, United States; ^9^Division of Cancer Prevention, National Cancer Institute (NCI), National Institutes of Health (NIH), Bethesda, MD, United States

**Keywords:** aging, health trajectories, healthspan, resilience, cardiovascular disease, neurological disorders, cancer

## Introduction

Many cultures throughout history have pursued the quest to improve longevity (1). Scientific advances, implementation in public health, and the use of vaccines and antibiotics have enhanced life expectancy over the last century (2). These interventions have reduced mortality but may have led to a concomitant rise in age-related multimorbidities (MM). Therefore, intervention initiatives need to incorporate the expanded goals of preventing age-related decline and extending healthspan—the period of life spent in good health and free from chronic diseases and disabilities (3). At its most essential, aging can be considered to result from impaired regulation of homeostasis, with a diminished ability to repair damage to critical molecular-cellular systems, a gradual decline in physiological functions, and accumulation of dysregulated and senescent cells over time. As people age, their immune systems become less resilient, leading to increased vulnerability to diseases and potentially contributing to the aging process. Resilience—the capacity to resist, adapt, recover, or grow in response to challenges—is believed to decrease with age and the development of age-related conditions (4). This definition of resilience for living systems adopted by the trans-NIH Resilience Working Group is relevant across multiple domains including environmental, community, and individual dimensions including genetic, molecular, cellular, physiological, psychological, and behavioral components. Immune resilience, the ability to maintain or regain optimal health during and after an infection can be indicative of an individual's overall health and aging trajectory. Those who can maintain or quickly return to this optimal state are likely to have a more favorable aging process (5). Behavioral and social factors can also impede or support the adoption of preventive strategies that increase resilience. An enhanced understanding of aging processes and resilience factors could facilitate strategies focused on improving early detection and intervention with the aim to delay the onset of age-related conditions, mitigating their severity, decreasing morbidity and frailty, and fostering healthier aging trajectories (6, 7).

Aging is linked to increased vulnerability to challenges contributing to aging-associated chronic diseases, such as cancer, cardiovascular diseases (CVD), neurodegenerative disorders, pulmonary conditions, and frailty. Preventing these or delaying their onset would improve the quality of life of our increasingly aging population. Identifying factors that promote healthy aging and preserve functional abilities and well-being has become a priority as the world's population ages. Understanding the commonalities and differences of the biological pathways involved in natural aging and age-related diseases is critical to influencing resilience outcomes, promoting health, and effective disease management or prevention (8).

**Figure 1:**
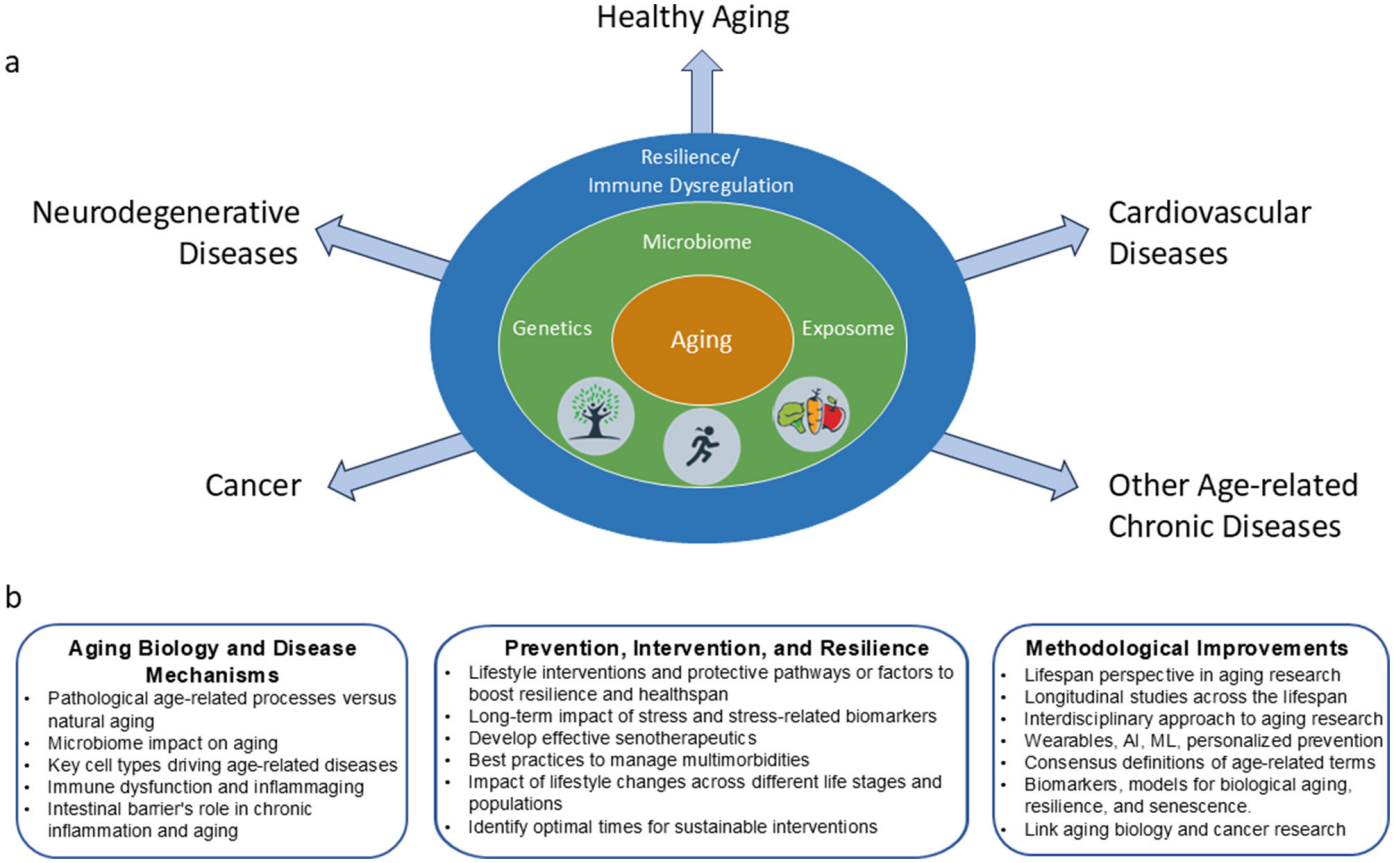
Factors that contribute to aging trajectories and possible research opportunity areas to address age-related diseases. **(a)** The interaction of complex factors such as genetics and lifestyle influences converge to drive an organism toward healthy aging or to the development of age-related diseases. **(b)** Research opportunity areas identified during the workshop. Increased understanding and collaborations across various expertise areas are needed to enhance health span and prevent or moderate the development of age-related disorders.

This manuscript summarizes knowledge gaps and current barriers emerging from an NIH workshop organized on the topic of: “Health and Aging Trajectories: Shared and Competing Risks and Resiliencies for Chronic Diseases Associated with Aging” (9). It also discusses novel research opportunities, ongoing efforts to address these gaps, and strategies for future research (Figure 1). We highlight the need for multi-disciplinary, collaborative efforts to develop interventions that enhance resilience and prevent chronic diseases, extend the healthy lifespan, and improve quality of life.

## The intricacies of competing and shared risks in aging trajectories

Unlike chronological aging, evenly measured in all individuals, biological aging varies among and within individuals leading to different aging trajectories. A complex interplay between inherent genetic factors and a range of external and lifestyle factors impact the course of biological aging and the onset of age-related chronic diseases (8). As we age, we encounter shared and competing risks that lead to multiple aging trajectories and influence health outcomes (10–12). These risks include genetics and the exposome—the lifetime exposure to internal factors and external environmental influences such as pollution. Critical psychosocial and lifestyle factors include diet, exercise, sleep patterns, and stress levels. Different groups of individuals have different pathways of age-associated molecular changes. Additionally, many age-related conditions share risk factors that often coexist as MM, requiring simultaneous management in individuals (13). MM prevalence presents differently in the general population (14). The COVID-19 pandemic highlighted the variable risk for cognitive impairments in older adults with MM (15). Furthermore, some groups exhibit a higher incidence of neurodegenerative diseases (16, 17). Despite adverse exposures, some people maintain healthier aging trajectories, providing complexity to the role of lifestyle and genetic factors against cognitive decline and age-related diseases. Other factors include sedentarism, unhealthy diets high in processed foods and saturated fats, smoking, excessive alcohol consumption, and chronic stress, which significantly increase the likelihood of developing age-related diseases. Older adults with limited social networks are more likely to experience poor health outcomes, including accelerated cognitive decline and higher mortality rates. Strategies to promote social connectivity, such as community engagement programs and digital tools, are increasingly recognized as important components of healthy aging. It is essential to ensure representation of older adults in clinical trials to better understand diverse healthspan pathways. These findings highlight the complex interplay of genetic predispositions on early onset of disease in susceptible populations (11). The circumstances mentioned create distinct aging patterns identified as “ageotypes”, that can contribute to our ability to measure and monitor variable aging trajectories (18). Understanding mechanisms and trajectories will allow us to identify novel approaches that can help slow or even reverse genetic, molecular and cellular hallmarks of aging and extend healthspan and longevity (7, 10–12, 19).

To develop a better understanding of diverse healthspan pathways, it is essential that longitudinal clinical trials strive for population-based recruitment and enrollment. The Baltimore Longitudinal Study of Aging, the longest running study of aging in the United States, has contributed to our knowledge of normal aging processes by looking at multiple phenotypic parameters uncovering a complex, heterogeneous pattern of aging trajectories (20, 21). The Danish Disease Trajectory Browser (22) provides a new perspective on disease progression patterns that can reveal associations between complex multimorbidities and potentially identify preventive strategies for chronic diseases (23). To promote interdisciplinary research on determinants and dynamics of within-person aging-related changes in cognitive and physical capabilities, health, personality, and well-being, the Integrative Analysis of Longitudinal Studies of Aging and Dementia (IALSA) research network provides access to meta-data from over 100 studies (24, 25).

## Biological processes promoting divergent aging trajectories

Several biological processes—briefly listed below—have been shown to contribute to aging trajectories and are inextricably linked to the emergence of age-related chronic diseases (7). Some of these processes affect cells and tissues across the whole body; others are specific to particular tissues and physiological functions.

### Cellular senescence

Cellular senescence is a fundamental aspect of aging in which a growing number of cells with increasingly anti-apoptotic mutations continue to exist within the tissue ecosystem but cease to divide (26, 27). Senescent cells accumulate within all tissues impairing cellular functions through production of various proinflammatory molecules (termed senescence-associated secretory phenotype). Senescent cells contribute to a range of age-related conditions that result from disruptions in normal cell functions across various bodily systems (28). Within the central nervous system, senescent cells contribute to structural brain changes and cognitive decline (29). They can also tilt the scale toward the development of cancer (30–32). Cellular senescence is also associated with reduced resilience and a shortened lifespan, and represents a potential therapeutic target to reduce severity and morbidity in COVID-19 infections (33). Consequently, there is a burgeoning interest in the development of senolytics, a category of drugs aimed at targeting and eliminating senescent cells. This therapeutic strategy holds the potential to mitigate age-related chronic diseases, thereby enhancing resilience and extending healthspan (34–38). The exploration of senolytics has yielded promising results, though it remains premature to draw definitive conclusions (39, 40). Enhancing the specificity of these compounds and optimizing treatment protocols, including dosage, is critical to mitigate adverse effects. Interestingly, senolytics have been identified in natural compounds, indicating future potential approaches for nutraceuticals in managing aging-related diseases (41).

### Malignancy

The transformation from normal to malignant cells, as outlined by the somatic evolution theory, establishes a connection between aging and the development of cancer (42). As we age, our DNA repair mechanisms become less efficient, leading to an accumulation of mutations. These mutations, combined with changes in the immune system, can contribute to the onset of cancer, atherosclerosis, and other chronic diseases. Aging and cancer share several key features, including genomic instability, alterations in metabolism, changes in telomeres, and cell senescence, all of which present potential targets for therapeutic intervention (43–45).

### Hematopoietic dysregulation and immune system dysfunction

Disruptions of generation and function in both innate and adaptive immune cells, coincides with the manifestation of aging-associated morbidities. At the generation step, hematopoiesis is shifted toward myelopoiesis at the expense of lymphopoiesis in the bone marrow, reducing the output of lymphocytes (46–48). Together with thymic involution and a life-long antigen exposure, naïve B cells and T cells are reduced, and antigen-experienced memory B cells and T cell subsets are increased in the periphery (49–52), thereby limiting responses to infections, tissue impairments, and cancer. Aging-associated B cells (53, 54) inhibit survival of pro-B cells in the bone marrow (55) and cause polarization of peripheral Th17 and Th1 cells (56), while aging-activated innate B1 B cells promote insulin resistance in older adults (57) and induce potentially autoimmune CD8+ T cells (58). Myeloid cells, such as monocytes and macrophages, show impaired phagocytosis, thus inefficiently clearing apoptotic cells and pathogens in aging (59, 60). The dysregulation as well as decline in immune function (termed immune senescence) increases in advanced age, contributing to the increased incidence of CVD, cancer, and degenerative conditions (61). Additionally, clonal hematopoiesis, which is characterized by the accumulation of somatic mutations in hematopoietic stem cells, has also been implicated in the onset of various age-related diseases (62–65).

### Endothelial dysfunction

Endothelial dysfunction, a key aspect of aging-related metabolic shifts, leads to impaired vascular tone, pro-thrombotic and pro-inflammatory states, contributing to widespread vascular and organ decline (66). This dysfunction underpins the progression of CVD, cancer, and degenerative conditions like vascular dementia (67–71). Age-driven vascular changes in the brain, which are more frequently observed in women, can diminish cognitive function and brain volume, potentially marking early signs of brain aging (72).

### Dysbiosis

Accumulating evidence demonstrates the gut microbiome's role in age-related changes in metabolism, digestion, immunity, mood, and cognition, influencing individuals' health. Aging can shift the microbiome toward pro-inflammatory bacteria, affecting metabolism, weakening intestinal integrity, and leading to low-grade inflammation (73). This microbiome evolution, linked to brain health via the gut-brain axis, may contribute to neurodegenerative diseases (74). Given its sensitivity to diet, medication, and environment, influencing the microbiome offers a potential strategy for preventing and treating age-related conditions (75–78).

### Immune dysregulation

Both innate and adaptive immune cell compartments, impairing their function and increasing chronic low-levels of harmful inflammation is defined as inflammaging (47, 48, 79). As such, directly or by contributing to inflammaging, the dysregulated immune cells in turn further age-related pathologies and diseases. Dysbiosis and activation of myeloid cells inhibit lymphopoiesis in the bone marrow, while accumulation of potentially pathogenic B cells contributes to increased insulin resistance in aging (57) and neurodegeneration (80).

### Neuropathology

Alzheimer's Disease and related dementias (ADRD) are characterized by changes in neuronal and perineuronal protein structure and function. The most notable and long-studied neuropathology includes amyloid plaques and hyperphosphorylated tau in neurofibrillary tangles. Implementing interventions earlier in ADRD progression, such as in those with mild cognitive impairment (MCI), could potentially reduce or prevent the progression of cognitive decline and dementia (81, 82). Utilizing biomarkers like plasma amyloid and tau alongside neuroimaging can reveal the neurocognitive impacts of aging, concomitant with the contribution of various risk factors such as hypertension, genetics, and lifestyle on health outcomes (83, 84).

### Psychogenic aging

Psychological factors—including responses to stress and resilience—contribute to healthspan and lifespan. For example, childhood experiences can have an impact on chronic diseases and early mortality. Brain-body circuits play a pivotal role in mediating interactions between environment, lifestyle, and aging. The body's cellular responses to stress begin in the nervous system, with the release of neurotransmitters and the stimulation of neuroendocrine pathways (e.g. the hypothalamic-pituitary-adrenal axis) which have the potential to influence various biological aging processes. Stress-related chemokines can trigger the mobilization of immune cells from the bone marrow and can lead to neuroinflammation (85). The identification of biomarkers associated with the psychogenic aging could reveal the profound effects of depression and loneliness on age-related morbidity, enhancing our comprehension of psychosocial resilience and its contribution to longevity and healthspan.

## Discussion: shared and competing risks to improve aging trajectories

A pressing research priority in the field of aging is the identification, stratification, and management of shared and distinct disease risks. Understanding how these risks interplay and how they can be mitigated is critical to extend healthspan. Regular physical activity, a Mediterranean-style diet rich in fruits, vegetables, whole grains, and omega-3 fatty acids, cognitive engagement, and stress management have shown promise in delaying or preventing cognitive decline, reducing cardiovascular risk, and improving overall health. Pleiotropic interventions—those producing multiple positive effects on health—represent an efficient path to improve health outcomes (86–89). For example, weight loss has shown wide-ranging benefits, improving health outcomes in patients with anxiety, depression, rheumatoid arthritis, diabetes, hypertension, and cancer (90, 91). Importantly, applying weight reduction strategies early in childhood and adolescence can potentially delay the onset of multiple chronic conditions later in life, highlighting the importance of timing for optimal intervention (92, 93). Exercise similarly demonstrates strong evidence for both slowing disease progression and preventing chronic conditions (93).

Mental stimulation and physical activity have also been shown to reduce the risk of MCI (82), while improved sleep duration is linked to reduction in inflammatory cytokines, mental health issues, and other outcomes crucial for healthy aging (94). Additional factors such as access to health care, social support, adherence to medications, reducing environmental pollution, and practices like mindfulness meditation have proven effective in improving risk factors and long-term health outcomes (95–100). Combining multiple effective interventions, and identifying critical life stages for testing and intervention hold immense promise for advancing preventive strategies and promoting better aging trajectories.

The evaluation of individual health and disease status requires detailed, longitudinal measurements. Technological advancements in artificial intelligence (AI) such as machine learning (ML) and large language models (LLMs) offer transformative opportunities for personalized care, early disease detection, and targeted interventions (101). Wearable devices, which provide continuous health monitoring, enable a deeper understanding of the interplay between genetics, environment, and lifestyle. These tools facilitate the development of personalized preventive and treatment strategies for age-related chronic diseases (102).

Aging has been considered as either a disease or a normal biological process. This classification has driven strategies such as testing drugs intended for age-related diseases as an indirect means of addressing aging. Alternative strategies need to be carefully evaluated to avoid potential health risks. There is an urgent need for a standardized definition of normal aging vs. age-related chronic diseases, along with associated biomarkers, and the creation of innovative models for studying biological aging. These measures are crucial for establishing reliable and effective intervention strategies (1). The field of geroscience seeks to understand how factors impacting common cellular and molecular processes lead to physiological dysfunction and chronic diseases. It aims to identify novel approaches to help slow down or even reverse genetic, molecular, and cellular hallmarks of aging and extend healthspan and longevity (10, 21, 103–105).

A forward-looking research agenda must integrate multidisciplinary approaches, incorporating advanced knowledge of genomics, other omics, and the exposome. Environmental exposures can interfere with gene expression pathways, biochemical traits, and physiological functions. Individual psychological traits, cognitive processes, and emotional responses also influence the ability to cope with challenges and adopt healthy behaviors (21, 104, 105). Closing the gap between lifespan and healthspan requires the creation of innovative strategies to make age-related diseases more predictable, preventable, and manageable. Gaining insights into the essential elements that maintain balance throughout life and the factors that disrupt this balance could lead to the identification of novel diagnostic markers and treatment targets (106, 107). Clinical longitudinal studies will help identify critical periods for effective interventions. Furthermore, compiling comprehensive and diverse datasets through cutting-edge technologies will accelerate discoveries and their clinical applications. Achieving the ambitious research goals set forth in this workshop demands interdisciplinary collaborations to address the complexities of aging and improve early disease detection. It is equally critical to prioritize the perspectives of patients in all phases of research. Finally, translating research findings from the laboratory into clinical practice will be pivotal in delivering tangible benefits to the aging population.

## References

[1] Murphy CT. How We Age: The Science of Longevity. Princeton, NJ: Princeton University Press. (2023). p. 14. 10.1515/9780691250335

[2] CDC. Mortality Trends in the United States, 1900–2018. National Center for Health Statistics. (2020). Available online at: https://www.cdc.gov/nchs/data-visualization/mortality-trends/index.htm (accessed March 9, 2024).

[3] Kaeberlein M. How Healthy Is the Healthspan Concept? GeroScience. (2018) 40:361–4. 10.1007/s11357-018-0036-9 30084059 PMC6136295

[4] Abadir PM Bandeen-Roche K Bergeman C Bennett D Davis D Kind A. An overview of the resilience world: Proceedings of the American Geriatrics Society and National Institute on Aging State of Resilience Science Conference. J Am Geriatr Soc. (2023) 71:2381–92. 10.1111/jgs.18388 37079440 PMC10523918

[5] Ahuja SK Manoharan MS Lee GC McKinnon LR Meunier JA Steri M. Immune resilience despite inflammatory stress promotes longevity and favorable health outcomes including resistance to infection. Nat Commun. (2023) 14:3286. 10.1038/s41467-023-38238-6 37311745 PMC10264401

[6] Hine C Yang J Zhang A Llarena N Link C. H_2_S Supplementation and Augmentation: Approaches for Healthy Aging. Hydrogen Sulfide: Chemical Biology Basics, Detection Methods, Therapeutic Applications, and Case Studies. Hoboken, NJ: John Wiley & Sons, Inc. (2022). p. 445–88.

[7] López-Otín C Blasco MA Partridge L Serrano M Kroemer G. The hallmarks of aging. Cell. (2013) 153:1194–217. 10.1016/j.cell.2013.05.039 23746838 PMC3836174

[8] Ferrucci L Gonzalez-Freire M Fabbri E Simonsick E Tanaka T Moore Z. Measuring biological aging in humans: a quest. Aging Cell. (2020) 19:e13080. 10.1111/acel.13080 31833194 PMC6996955

[9] NHLBI. Health and Aging Trajectories: Shared and Competing Risks and Resiliencies for Chronic Diseases Associated with Aging. (2023). Available online at: https://www.nhlbi.nih.gov/events/2023/health-and-aging-trajectories-shared-and-competing-risks-and-resiliencies-chronic (accessed February 27, 2024).

[10] NIA. Geroscience: The Intersection of Basic Aging Biology, Chronic Disease, and Health. Available online at: https://www.nia.nih.gov/research/dab/geroscience-intersection-basic-aging-biology-chronic-disease-and-health (accessed March 15, 2024). 10.1097/RNJ.0000000000000231 31083057

[11] Crimmins EM. Social hallmarks of aging: suggestions for geroscience research. Ageing Res Rev. (2020) 63:101136. 10.1016/j.arr.2020.101136 32798771 PMC7530044

[12] Epel ES. The geroscience agenda: toxic stress, hormetic stress, and the rate of aging. Ageing Res Rev. (2020) 63:101167. 10.1016/j.arr.2020.101167 32979553 PMC7520385

[13] Salive ME. Multimorbidity in older adults. Epidemiol Rev. (2013) 35:75–83. 10.1093/epirev/mxs009 23372025

[14] Quiñones AR Newsom JT Elman MR Markwardt S Nagel CL Dorr DA. Racial and ethnic differences in multimorbidity changes over time. Med Care. (2021) 59:402–9. 10.1097/MLR.0000000000001527 33821829 PMC8024615

[15] Davis HE McCorkell L Vogel JM Topol EJ. Long COVID: major findings, mechanisms and recommendations. Nat Rev Microbiol. (2023) 21:133–46. 10.1038/s41579-022-00846-2 36639608 PMC9839201

[16] Nuytemans K Rajabli F Jean-Francois M Kurup JT Adams LD Starks TD. Genetic analyses in multiplex families confirms chromosome 5q35 as a risk locus for Alzheimer's disease in individuals of African ancestry. Neurobiol Aging. (2024) 133:125–33. 10.1016/j.neurobiolaging.2023.10.010 37952397 PMC11131578

[17] Chen N Caruso C Alonso A Derebail VK Kshirsagar AV Sharrett AR. Association of sickle cell trait with measures of cognitive function and dementia in African Americans. eNeurologicalSci. (2019) 16:100201. 10.1016/j.ensci.2019.100201 31384675 PMC6661502

[18] Ahadi S Zhou W Schüssler-Fiorenza Rose SM Sailani MR Contrepois K Avina M. Personal aging markers and ageotypes revealed by deep longitudinal profiling. Nat Med. (2020) 26:83–90. 10.1038/s41591-019-0719-5 31932806 PMC7301912

[19] Moffitt TE. Behavioral and social research to accelerate the geroscience translation agenda. Ageing Res Rev. (2020) 63:101146. 10.1016/j.arr.2020.101146 32814128 PMC7894048

[20] Kuo P-L Schrack JA Shardell MD Levine M Moore AZ An Y. A roadmap to build a phenotypic metric of ageing: insights from the Baltimore Longitudinal Study of Aging. J Intern Med. (2020) 287:373–94. 10.1111/joim.13024 32107805 PMC7670826

[21] Kuo P-L Schrack JA Levine ME Shardell MD Simonsick EM Chia CW. Longitudinal phenotypic aging metrics in the Baltimore Longitudinal Study of Aging. Nature Aging. (2022) 2:635–43. 10.1038/s43587-022-00243-7 36910594 PMC9997119

[22] Siggaard T Reguant R Jørgensen IF Haue AD Lademann M Aguayo-Orozco A. Disease trajectory browser for exploring temporal, population-wide disease progression patterns in 7.2 million Danish patients. Nat Commun. (2020) 11:4952. 10.1038/s41467-020-18682-4 33009368 PMC7532164

[23] Jensen AB Moseley PL Oprea TI Ellesøe SG Eriksson R Schmock H. Temporal disease trajectories condensed from population-wide registry data covering 6.2 million patients. Nat Commun. (2014) 5:4022. 10.1038/ncomms5022 24959948 PMC4090719

[24] Hofer SM Piccinin AM. Toward an integrative science of life-span development and aging. J Gerontol Series B. (2010) 65B:269–78. 10.1093/geronb/gbq017 20237144 PMC2853604

[25] Research M. Integrative Analysis of Longitudinal Studies of Aging and Dementia: The Research Institute of the McGill University Health Centre (RI MUHC). (2021). Available onlin at: https://www.maelstrom-research.org/network/ialsa (accessed December 20, 2024).

[26] Childs BG Gluscevic M Baker DJ Laberge R-M Marquess D Dananberg J. Senescent cells: an emerging target for diseases of ageing. Nat Rev Drug Discovery. (2017) 16:718–35. 10.1038/nrd.2017.116 28729727 PMC5942225

[27] Lee P Benz C Blood P Borner K Campisi J Chen F. NIH SenNet Consortium to map senescent cells throughout the human lifespan to understand physiological health. Nature Aging. (2022) 2:0160. 10.20944/preprints202207.0160.v1 PMC10019484 36936385

[28] Bloom SI Islam MT Lesniewski LA Donato AJ. Mechanisms and consequences of endothelial cell senescence. Nat Rev Cardiol. (2023) 20:38–51. 10.1038/s41569-022-00739-0 35853997 PMC10026597

[29] Sikora E Bielak-Zmijewska A Dudkowska M Krzystyniak A Mosieniak G Wesierska M. Cellular senescence in brain aging. Front Aging Neurosci. (2021) 13:646924. 10.3389/fnagi.2021.646924 33732142 PMC7959760

[30] Schmitt CA Wang B Demaria M. Senescence and cancer — role and therapeutic opportunities. Nat Rev Clini Oncol. (2022) 19:619–36. 10.1038/s41571-022-00668-4 36045302 PMC9428886

[31] Faget DV Ren Q Stewart SA. Unmasking senescence: context-dependent effects of SASP in Cancer. Nat Rev Cancer. (2019) 19:439–53. 10.1038/s41568-019-0156-2 31235879

[32] Faget DV Stewart SA. Stress response regulates cancer fibroblasts. Nat Cell Biol. (2022) 24:812–4. 10.1038/s41556-022-00930-y 35654838

[33] Schmitt CA Tchkonia T Niedernhofer LJ Robbins PD Kirkland JL Lee S. COVID-19 and cellular senescence. Nat Rev Immunol. (2023) 23:251–63. 10.1038/s41577-022-00785-2 36198912 PMC9533263

[34] Calabrò A Accardi G Aiello A Caruso C Galimberti D Candore G. Senotherapeutics to counteract senescent cells are prominent topics in the context of anti-ageing strategies. Int J Mol Sci. (2024) 25:1792. 10.3390/ijms25031792 38339070 PMC10855240

[35] Justice JN Nambiar AM Tchkonia T LeBrasseur NK Pascual R Hashmi SK. Senolytics in idiopathic pulmonary fibrosis: results from a first-in-human, open-label, pilot study. EBioMedicine. (2019) 40:554–63. 10.1016/j.ebiom.2018.12.052 30616998 PMC6412088

[36] Zhang L Zhao J Mu X McGowan SJ Angelini L O'Kelly RD. Novel small molecule inhibition of IKK/NF-κb activation reduces markers of senescence and improves healthspan in mouse models of aging. Aging Cell. (2021) 20:e13486. 10.1111/acel.13486 34734460 PMC8672781

[37] Narasimhan A Flores RR Camell CD Bernlohr DA Robbins PD Niedernhofer LJ. Cellular senescence in obesity and associated complications: a new therapeutic target. Curr Diab Rep. (2022) 22:537–48. 10.1007/s11892-022-01493-w 36239841 PMC10123542

[38] Islam MT Hall SA Dutson T Bloom SI Bramwell RC Kim J. Endothelial cell-specific reduction in mTor ameliorates age-related arterial and metabolic dysfunction. Aging Cell. (2024) 23:e14040. 10.1111/acel.14040 38017701 PMC10861194

[39] Suda M Paul KH Minamino T Miller JD Lerman A Ellison-Hughes GM. Senescent cells: a therapeutic target in cardiovascular diseases. Cells. (2023) 12:1296. 10.3390/cells12091296 37174697 PMC10177324

[40] Gonzales MM Garbarino VR Kautz TF Palavicini JP Lopez-Cruzan M Dehkordi SK. Senolytic therapy in mild Alzheimer's disease: a phase 1 feasibility trial. Nat Med. (2023) 29:2481–8. 10.1038/s41591-023-02543-w 37679434 PMC10875739

[41] Gurău F Baldoni S Prattichizzo F Espinosa E Amenta F Procopio AD. Anti-senescence compounds: a potential nutraceutical approach to healthy aging. Ageing Res Rev. (2018) 46:14–31. 10.1016/j.arr.2018.05.001 29742452

[42] Marongiu F DeGregori J. The sculpting of somatic mutational landscapes by evolutionary forces and their impacts on aging-related disease. Mol Oncol. (2022) 16:3238–58. 10.1002/1878-0261.13275 35726685 PMC9490148

[43] Braithwaite D Anton S Mohile S DeGregori J Gillis N Zhou D. Cancer and aging: a call to action. Aging and Cancer. (2022) 3:87–94. 10.1002/aac2.12055 36188489 PMC9521708

[44] Zabransky DJ Jaffee EM Weeraratna AT. Shared genetic and epigenetic changes link aging and cancer. Trends Cell Biol. (2022) 32:338–50. 10.1016/j.tcb.2022.01.004 35144882 PMC10202213

[45] Fane M Weeraratna AT. How the ageing microenvironment influences tumour progression. Nat Rev Cancer. (2020) 20:89–106. 10.1038/s41568-019-0222-9 31836838 PMC7377404

[46] Roeder I Horn K Sieburg H-B Cho R Muller-Sieburg C Loeffler M. Characterization and quantification of clonal heterogeneity among hematopoietic stem cells: a model-based approach. Blood. (2008) 112:4874–83. 10.1182/blood-2008-05-155374 18809760 PMC2597595

[47] Cho RH Sieburg HB Muller-Sieburg CE. A new mechanism for the aging of hematopoietic stem cells: aging changes the clonal composition of the stem cell compartment but not individual stem cells. Blood. (2008) 111:5553–61. 10.1182/blood-2007-11-123547 18413859 PMC2424153

[48] Kuranda K Vargaftig J. de la Rochere P Dosquet C Charron D Bardin F. Age-related changes in human hematopoietic stem/progenitor cells. Aging Cell. (2011) 10:542–6. 10.1111/j.1474-9726.2011.00675.x 21418508

[49] Koch S Larbi A Derhovanessian E Özcelik D Naumova E Pawelec G. Multiparameter flow cytometric analysis of CD4 and CD8 T cell subsets in young and old people. Immunity Ageing. (2008) 5:6. 10.1186/1742-4933-5-6 18657274 PMC2515281

[50] Lerner A Yamada T Miller RA. Pgp-1^hi^ T lymphocytes accumulate with age in mice and respond poorly to concanavalin A. Eur J Immunol. (1989) 19:977–82. 10.1002/eji.1830190604 2666144

[51] Guerrettaz LM Johnson SA Cambier JC. Acquired hematopoietic stem cell defects determine B-cell repertoire changes associated with aging. Proc Nat Acad Sci. (2008) 105:11898–902. 10.1073/pnas.0805498105 18697924 PMC2515225

[52] Cancro MP Allman DM. Connecting the dots: revealing the interactions of lymphocyte development and homeostasis in the immunobiology of aging. Semin Immunol. (2005) 17:319–20. 10.1016/j.smim.2005.05.017 15990334

[53] Rubtsov AV Rubtsova K Fischer A Meehan RT Gillis JZ Kappler JW. Toll-like receptor 7 (TLR7)–driven accumulation of a novel CD11c^+^ B-cell population is important for the development of autoimmunity. Blood. (2011) 118:1305–15. 10.1182/blood-2011-01-331462 21543762 PMC3152497

[54] Hao Y O'Neill P Naradikian MS Scholz JL Cancro MP. A B-cell subset uniquely responsive to innate stimuli accumulates in aged mice. Blood. (2011) 118:1294–304. 10.1182/blood-2011-01-330530 21562046 PMC3152496

[55] Ratliff M Alter S Frasca D Blomberg BB Riley RL. In senescence, age-associated B cells secrete TNFα and inhibit survival of B-cell precursors. Aging Cell. (2013) 12:303–11. 10.1111/acel.12055 23410004 PMC3716274

[56] Tomihara K Shin T Hurez VJ Yagita H Pardoll DM Zhang B. Aging-associated B7-DC^+^ B cells enhance anti-tumor immunity via Th1 and Th17 induction. Aging Cell. (2012) 11:128–38. 10.1111/j.1474-9726.2011.00764.x 22044484 PMC3683836

[57] Bodogai M O'Connell J Kim K Kim Y Moritoh K Chen C. Commensal bacteria contribute to insulin resistance in aging by activating innate B1a cells. Sci Transl Med. (2018) 10:eaat4271. 10.1126/scitranslmed.aat4271 30429354 PMC6445267

[58] Lee-Chang C Bodogai M Moritoh K Chen X Wersto R Sen R. Aging converts innate B1a cells into potent CD8+ T cell inducers. J Immunol. (2016) 196:3385–97. 10.4049/jimmunol.1502034 26983789 PMC4821757

[59] Hearps AC Martin GE Angelovich TA Cheng W-J Maisa A Landay AL. Aging is associated with chronic innate immune activation and dysregulation of monocyte phenotype and function. Aging Cell. (2012) 11:867–75. 10.1111/j.1474-9726.2012.00851.x 22708967

[60] Plowden J Renshaw-Hoelscher M Engleman C Katz J Sambhara S. Innate immunity in aging: impact on macrophage function. Aging Cell. (2004) 3:161–7. 10.1111/j.1474-9728.2004.00102.x 15268749

[61] Larbi A Franceschi C Mazzatti D Solana R Wikby A Pawelec G. Aging of the immune system as a prognostic factor for human longevity. Physiology. (2008) 23:64–74. 10.1152/physiol.00040.2007 18400689

[62] Miller PG Qiao D Rojas-Quintero J Honigberg MC Sperling AS Gibson CJ. Association of clonal hematopoiesis with chronic obstructive pulmonary disease. Blood. (2022) 139:357–68. 10.1182/blood.2021013531 34855941 PMC8777202

[63] Saadatagah S Uddin MM Weeks LD Niroula A Ru M Takahashi K. Clonal hematopoiesis risk score and all-cause and cardiovascular mortality in older adults. JAMA Netw Open. (2024) 7:e2351927-e. 10.1001/jamanetworkopen.2023.51927 38231513 PMC10794939

[64] Bouzid H Belk JA Jan M Qi Y Sarnowski C Wirth S. Clonal hematopoiesis is associated with protection from Alzheimer's disease. Nat Med. (2023) 29:1662–70. 10.1038/s41591-023-02397-2 37322115 PMC10353941

[65] Challen GA Goodell MA. Clonal hematopoiesis: mechanisms driving dominance of stem cell clones. Blood. (2020) 136:1590–8. 10.1182/blood.2020006510 32746453 PMC7530644

[66] Widlansky ME Gokce N Keaney JF Vita JA. The clinical implications of endothelial dysfunction. J Am Coll Cardiol. (2003) 42:1149–60. 10.1016/S0735-1097(03)00994-X 14522472

[67] Rajendran P Rengarajan T Thangavel J Nishigaki Y Sakthisekaran D Sethi G. The vascular endothelium and human diseases. Int J Biol Sci. (2013) 9:1057–69. 10.7150/ijbs.7502 24250251 PMC3831119

[68] Wardlaw JM Smith C Dichgans M. Small vessel disease: mechanisms and clinical implications. Lancet Neurol. (2019) 18:684–96. 10.1016/S1474-4422(19)30079-1 31097385

[69] Donato AJ Machin DR Lesniewski LA. Mechanisms of dysfunction in the aging vasculature and role in age-related disease. Circ Res. (2018) 123:825–48. 10.1161/CIRCRESAHA.118.312563 30355078 PMC6207260

[70] Lakatta EG Levy D. Arterial and cardiac aging: major shareholders in cardiovascular disease enterprises. Circulation. (2003) 107:139–46. 10.1161/01.CIR.0000048892.83521.58 12515756

[71] Terwoord JD Beyer AM Gutterman DD. Endothelial dysfunction as a complication of anti-cancer therapy. Pharmacol Ther. (2022) 237:108116. 10.1016/j.pharmthera.2022.108116 35063569 PMC9294076

[72] Torres-Espin A Radabaugh HL Treiman S Fitzsimons SS Harvey D Chou A., Sexually dimorphic differences in angiogenesis markers are associated with brain aging trajectories in humans. Sci. Transl. Med. (2024) 16:eadk3118. 10.1126/scitranslmed.adk3118 39602511 PMC12092094

[73] Wu Y-L Xu J Rong X-Y Wang F Wang H-J Zhao C. Gut microbiota alterations and health status in aging adults: from correlation to causation. Aging Med. (2021) 4:206–13. 10.1002/agm2.12167 34553118 PMC8444961

[74] Khan R Di Gesù CM Lee J McCullough LD. The contribution of age-related changes in the gut-brain axis to neurological disorders. Gut Microbes. (2024) 16:2302801. 10.1080/19490976.2024.2302801 38237031 PMC10798364

[75] Forero-Rodríguez J Zimmermann J Taubenheim J Arias-Rodríguez N Caicedo-Narvaez JD Best L. Changes in bacterial gut composition in Parkinson's disease and their metabolic contribution to disease development: a gut community reconstruction approach. Microorganisms. (2024) 12:325. 10.3390/microorganisms12020325 38399728 PMC10893096

[76] Ye C Li Z Ye C Yuan L Wu K Zhu C. Association between gut microbiota and biological aging: a two-sample Mendelian randomization study. Microorganisms. (2024) 12:370. 10.3390/microorganisms12020370 38399774 PMC10891714

[77] Vivarelli S Salemi R Candido S Falzone L Santagati M Stefani S. Gut microbiota and cancer: from pathogenesis to therapy. Cancers. (2019) 11:38. 10.3390/cancers11010038 30609850 PMC6356461

[78] Marinos G Hamerich Inga K Debray R Obeng N Petersen C Taubenheim J. Metabolic model predictions enable targeted microbiome manipulation through precision prebiotics. Microbiology Spectrum. (2024) 12:e01144–23. 10.1128/spectrum.01144-23 38230938 PMC10846184

[79] Biragyn A Ferrucci L. Gut dysbiosis: a potential link between increased cancer risk in ageing and inflammaging. Lancet Oncol. (2018) 19:e295–304. 10.1016/S1470-2045(18)30095-0 29893261 PMC6047065

[80] Kim K Wang X Ragonnaud E Bodogai M Illouz T DeLuca M. Therapeutic B-cell depletion reverses progression of Alzheimer's disease. Nat Commun. (2021) 12:2185. 10.1038/s41467-021-22479-4 33846335 PMC8042032

[81] Krell-Roesch J Syrjanen JA Vassilaki M Machulda MM Mielke MM Knopman DS. Quantity and quality of mental activities and the risk of incident mild cognitive impairment. Neurology. (2019) 93:e548–e58. 10.1212/WNL.0000000000007897 31292224 PMC6710000

[82] Geda YE. Mild cognitive impairment in older adults. Curr Psychiatry Rep. (2012) 14:320–7. 10.1007/s11920-012-0291-x 22773365 PMC3963488

[83] Bilgel M An Y Walker KA Moghekar AR Ashton NJ Kac PR. Longitudinal changes in Alzheimer's-related plasma biomarkers and brain amyloid. Alzheimer's & *Dementia*. (2023) 19:4335–45. 10.1002/alz.13157 37216632 PMC10592628

[84] Hansson O. Biomarkers for neurodegenerative diseases. Nat Med. (2021) 27:954–63. 10.1038/s41591-021-01382-x 34083813

[85] Faria M Ganz A Galkin F Zhavoronkov A Snyder M. Psychogenic aging: a novel prospect to integrate psychobiological hallmarks of aging. Transl Psychiatry. (2024) 14:226. 10.1038/s41398-024-02919-7 38816369 PMC11139997

[86] Lui DTW Tan KCB. High-density lipoprotein in diabetes: structural and functional relevance. J Diabet Investigat. (2024) 15:805–16. 10.1111/jdi.14172 38416054 PMC11215696

[87] Pisanu C Congiu D Meloni A Paribello P Patrinos GP Severino G. Dissecting the genetic overlap between severe mental disorders and markers of cellular aging: identification of pleiotropic genes and druggable targets. Neuropsychopharmacology. (2024) 49:1033–41. 10.1038/s41386-024-01822-5 38402365 PMC11039620

[88] The Emerging Risk Factors Collaboration. Association of cardiometabolic multimorbidity with mortality. JAMA. (2015) 314:52–60. 10.1001/jama.2015.7008 26151266 PMC4664176

[89] Gaziano L Sun L Arnold M Bell S Cho K Kaptoge SK. Mild-to-moderate kidney dysfunction and cardiovascular disease: observational and Mendelian randomization analyses. Circulation. (2022) 146:1507–17. 10.1161/CIRCULATIONAHA.122.060700 36314129 PMC9662821

[90] Bradley T Campbell E Dray J Bartlem K Wye P Hanly G. Systematic review of lifestyle interventions to improve weight, physical activity and diet among people with a mental health condition. Syst Rev. (2022) 11:198. 10.1186/s13643-022-02067-3 36085250 PMC9462072

[91] Gray MS Judd SE Sloane R Snyder DC Miller PE Demark-Wahnefried W. Rural–urban differences in health behaviors and outcomes among older, overweight, long-term cancer survivors in the RENEW randomized control trial. Canc Causes Cont. (2019) 30:301–9. 10.1007/s10552-019-01141-x 30783858 PMC6459722

[92] Smith JD Fu E Kobayashi MA. Prevention and management of childhood obesity and its psychological and health comorbidities. Annu Rev Clin Psychol. (2020) 16:351–78. 10.1146/annurev-clinpsy-100219-060201 32097572 PMC7259820

[93] Barnes JN Pearson AG Corkery AT Eisenmann NA Miller KB. Exercise, arterial stiffness, and cerebral vascular function: potential impact on brain health. J Int Neuropsychol Soc. (2021) 27:761–75. 10.1017/S1355617721000394 33952365 PMC8496967

[94] Blake MJ Sheeber LB Youssef GJ Raniti MB Allen NB. Systematic review and meta-analysis of adolescent cognitive–behavioral sleep interventions. Clin Child Fam Psychol Rev. (2017) 20:227–49. 10.1007/s10567-017-0234-5 28331991

[95] Baicker K Taubman SL Allen HL Bernstein M Gruber JH Newhouse JP. The Oregon experiment — effects of Medicaid on clinical outcomes. N Engl J Med. (2013) 368:1713–22. 10.1056/NEJMsa1212321 23635051 PMC3701298

[96] Ruppar TM Conn F Russell CL. Medication adherence interventions for older adults: literature review. Res Theory Nurs Pract. (2008) 22:114–47. 10.1891/1541-6577.22.2.114 18578221

[97] Davidson KW McGinn T. Screening for social determinants of health: the known and unknown. JAMA. (2019) 322:1037–8. 10.1001/jama.2019.10915 31465095

[98] Barrett M Combs V Su JG Henderson K Tuffli M. Air Louisville: addressing asthma with technology, crowdsourcing, cross-sector collaboration, and policy. Health Aff. (2018) 37:525–34. 10.1377/hlthaff.2017.1315 29608361

[99] Hoffman L Hutt R Yi Tsui CK Zorokong K Marfeo E. Meditation-based interventions for adults with dementia: a scoping review. Am J Occup Therapy. (2020) 74:7403205010p1-p14. 10.5014/ajot.2020.037820 32365307

[100] Zhang D Lee EKP Mak ECW Ho CY Wong SYS. Mindfulness-based interventions: an overall review. Br Med Bull. (2021) 138:41–57. 10.1093/bmb/ldab005 33884400 PMC8083197

[101] Binder J Ursu O Bologa C Jiang S Maphis N Dadras S. Machine learning prediction and tau-based screening identifies potential Alzheimer's disease genes relevant to immunity. Communications Biology. (2022) 5:125. 10.1038/s42003-022-03068-7 35149761 PMC8837797

[102] Wanigatunga AA Liu F Wang H Urbanek JK An Y Spira AP. Daily physical activity patterns as a window on cognitive diagnosis in the Baltimore Longitudinal Study of aging (BLSA). Journal of Alzheimer's Disease. (2022) 88:459–69. 10.3233/JAD-215544 35599480 PMC12276952

[103] Janssen H Koekkoek LL Swirski FK. Effects of lifestyle factors on leukocytes in cardiovascular health and disease. Nat Rev Cardiol. (2024) 21:157–69. 10.1038/s41569-023-00931-w 37752350

[104] Walsh K Raghavachari N Kerr C Bick AG Cummings SR Druley T. Clonal hematopoiesis analyses in clinical, epidemiologic, and genetic aging studies to unravel underlying mechanisms of age-related dysfunction in humans. Front Aging. (2022) 3:841796. 10.3389/fragi.2022.841796 35821803 PMC9261374

[105] Gonzales MM Garbarino VR Pollet E Palavicini JP Kellogg DL Jr. Kraig E. Biological aging processes underlying cognitive decline and neurodegenerative disease. J Clini Investigat. (2022) 132: 8453. 10.1172/JCI158453 35575089 PMC9106343

[106] Moen JM Morrell CH Matt MG Ahmet I Tagirova S Davoodi M. Emergence of heartbeat frailty in advanced age I: perspectives from life-long EKG recordings in adult mice. GeroSci. (2022) 44:2801–30. 10.1007/s11357-022-00605-4 35759167 PMC9768068

[107] Volkova M Zhang Y Shaw AC Lee PJ. The role of Toll-like receptors in age-associated lung diseases. J Gerontol: Series A. (2012) 67A:247–53. 10.1093/gerona/glr226 22396470 PMC3297763

